# The role of acoustic signals for species recognition in redfronted lemurs (*Eulemur rufifrons*)

**DOI:** 10.1186/s12862-016-0677-1

**Published:** 2016-05-12

**Authors:** Hanitriniaina Rakotonirina, Peter M. Kappeler, Claudia Fichtel

**Affiliations:** Behavioral Ecology & Sociobiology Unit, German Primate Center, Göttingen, Germany; Department of Sociobiology/Anthropology, University of Göttingen, Göttingen, Germany

**Keywords:** *Eulemur rufifrons*, Species recognition, Acoustic signals, Mate choice, Genetic drift

## Abstract

**Background:**

Signals are essential for communication and play a fundamental role in the evolution and diversification of species. Olfactory, visual and acoustic species-specific signals have been shown to function for species recognition in non-human primates, but the relative contributions of selection for species recognition driven by sexual selection, natural selection, or genetic drift for the diversification of these signals remain largely unexplored. This study investigates the importance of acoustic signals for species recognition in redfronted lemurs (*Eulemur rufifrons*). We conducted playback experiments in both major populations of this species separated by several hundred kilometers: Kirindy Forest in the west and Ranomafana National Park in the east of Madagascar. The playback stimuli were composed of species-specific loud calls of *E. rufifrons*, three closely related species (*E. albifrons*, *E. fulvus* and *E. rufus*) and one genetically more distant species (*E. rubriventer*) that occurs in sympatry with eastern redfronted lemurs. We tested the ability of redfronted lemurs to discriminate conspecific from heterospecific loud calls by measuring the time spent looking towards the speaker after presentation of each loud call. We also tested the difference between female and male responses because loud calls may play a role in mate choice and the avoidance of heterospecific mating.

**Results:**

Redfronted lemurs in Kirindy Forest did not discriminate their own loud calls from those of *E. albifrons*, *E. fulvus* and *E. rufus*, but they discriminated loud calls of *E. rubriventer* from their own. The Ranomafana population was tested only with three playback stimuli (*E. rufifrons, E. albifrons, E. rubriventer*) and did not discriminate between their own loud calls and those of *E. albifrons* and *E. rubriventer*. The response of females and males to playbacks did not differ in both populations. However, subjects in Ranomafana National Park responded more strongly to playback stimuli from *E. rubriventer* than subjects in Kirindy Forest.

**Conclusions:**

We conclude that in both populations individuals were not able to discriminate between loud calls of closely related species living in allopatry and that responses to more distantly related congeners are likely to be modulated by experience. Subjects in Ranomafana paid more attention to loud calls of syntopic *E. rubriventer* in comparison to the Kirindy subjects, suggesting that experience is important in facilitating discrimination. Because acoustic and genetic distances among eulemurs are correlated, diversification in their acoustic signals might be the result of genetic drift.

**Electronic supplementary material:**

The online version of this article (doi:10.1186/s12862-016-0677-1) contains supplementary material, which is available to authorized users.

## Background

Signals are not only essential for conspecific communication, but also play an important role in the evolution and diversification of species [1–3]. Species-specific signals may evolve in response to different evolutionary pressures. First, such signals may represent the result of sexual selection if they function as a premating isolation mechanism [4], requiring the ability for species recognition in heterospecific receivers [5–9]. Based on the ability of an individual to discriminate between signals from its own and other species, species recognition is used in many different taxa to avoid costly interbreeding. This ability has been demonstrated in several taxa, such as bats using olfactory signals [10], fish using olfactory or visual signals [11, 12] and frogs, birds and mammals using acoustic signals [6, 13–15].

Second, species-specific signals can also be the result of natural selection through adaptations to local habitat conditions. For example, frogs (*Amolops tormotus*) living close to noisy streams shifted the frequency of their calls in the ultra-sound range to avoid masking of background noise of the stream [16]. In little greenbul (*Andropadus virens*) occurring in two different forest types (rainforest or ecotone forest), habitat-dependent selection has also been suggested to cause divergence of acoustic traits because songs of rainforest populations differ in spectral and temporal characteristics compared to those in the ecotone forest [17]. Finally, signal diversification may also be driven by cultural or genetic drift, where stochastic processes generate species-specific signals in the absence of selection [18]. For example, in greenish warblers (*Phylloscopus trochiloides*) and Neotropical singing mice (*Scotinomys teguina*, *S. xerampelinus*), diversification in songs was shown to be correlated with both geographic distance and genetic divergence, suggesting that differentiation in this signal were largely shaped by genetic drift [19, 20]. Although the ability to use signals for species recognition is widespread, the relative contributions of selection for species recognition driven by sexual selection, natural selection, or genetic drift for the diversification of species signals remain poorly understood.

Primates are an interesting taxon for studies of species recognition because they often occur in sympatry with other species, they inhabit a range of tropical habitats, and they exhibit social communication, relying on olfactory, visual and acoustic signals. Sexual selection has been suggested to have driven diversification of primate olfactory signals [21, 22], and species recognition based on olfactory cues has been demonstrated in true lemurs (*Eulemur sp*. [23]), bushbabies (*Galago sp*. [24]) and capuchin monkeys (*Cebus sp*. [25]). Interspecific variation in visual signals has also been suggested to function in species recognition among primates [26–28]. For example, in both New World monkeys (platyrrhines) and Old World monkeys (catarrhines), facial color complexity is positively related to the number of sympatric congeners [29, 30]. However, the evolution of facial pigmentation and hair length in platyrrhines was linked to ecological factors since these traits are strongly related to the geographical distribution of species [29].

Acoustic signals have also been suggested to represent a useful tool for species delimitation in several primate species, including lion tamarins (*Leontopithecus rosalia*, *L. chrysopygus* and *L. chrysomelas* [31]), crested gibbons (*Nomascus gabriellae* and *N. leucogenys* siki [32]) and lemurs (Lemuridae [33]). Even in closely related species, such as gibbons (*Nomascus nasutus*, *N. concolor*, *N. leucogenys*, *N. siki*, *N. annamensis* and *N. gabriellae* [34]), langurs (*Presbytis thomasi*, *P.potenziani siberu*, *P.comata comata* and all four subspecies of *P. melalophos* (*P. m. melalophos, P. m. mitrata, P.m.bicolor* and *P.m.sumatrana*) [35]), Decken’s and crowned sifakas (*Propithecus deckenii* and *P. coronatus* [36]), or in black lemurs (*Eulemur macaco* and *E. flavifrons* [37]), calls are characterized by species-specific acoustic structure. However, whether these differences between acoustic signals evolved in the context of species recognition and are used to discriminate between conspecifics and heterospecifics by the animals remains unknown. Moreover, whether call divergence has been driven by habitat adaptations, as in catarrhines [38], or is the result of stochastic processes, as in gibbons [34], or of sexual selection, as in orangutans (*Pongo sp*. [39]), is often also unknown.

Specific tests involving playback experiments to demonstrate that primates are able to discriminate heterospecific from conspecific calls have only rarely been conducted (e.g. in tarsiers, *Tarsius* spp. [40]; macaques, *(Macaca tonkeana*, *M. maurus*, *M. hecki* and *M. nigrescens)* [41]; gibbons, *Hylobates* spp. [15, 42] and mouse lemurs, *Microcebus* ssp.: [43]) and yielded variable results. For example, Nietsch and Kopp [40] found that *Tarsius spectrum* discriminated vocalizations of conspecifics and heterospecifics (Diane’s and Tongian tarsiers). Mitani [42] showed that agile gibbons (*Hylobates agilis*) responded similarly to conspecific songs from the local and allopatric populations but differentiated between those and allopatric heterospecific songs (*H. muelleri*). Finally, gray mouse lemurs (*Microcebus murinus*), which occur in sympatry with golden-brown mouse lemurs (*M. ravelobensis)* but in allopatry with Goodman’s mouse lemurs (*M. lehilahytsara)* responded stronger to conspecific than to heterospecific advertisement calls (essential in the context of reproduction) and, interestingly, stronger to calls of the allopatric than the sympatric species [43]. This result suggests that the spatial cohesiveness of species in sympatry led to species-specific divergence of acoustic signals to avoid costly hybridization [43]. Thus, primates are able to discriminate between conspecific and heterospecific calls, irrespective of whether they occur in sympatry or allopatry (indicating different diversification mechanisms of acoustic signals in different genera).

In this study, we investigated the ability of redfronted lemurs (*Eulemur rufifrons*) to discriminate between loud calls of allopatric and sympatric congeners. The endemic Malagasy genus *Eulemur* consists of 12 species occupying all major primary habitats in Madagascar. Seven species of the genus, formerly classified as the “*Eulemur fulvus* group” (*E. albifrons, E. cinereiceps, E. collaris, E. fulvus, E. rufifrons, E. rufus,* and *E. sanfordi*) are closely related and probably diverged only in the last million years [44]. Geographically, they are distributed in allopatric populations and the other species of the genus *Eulemur* (*E. coronatus, E. flavifrons, E. macaco, E. mongoz,* and *E. rubriventer*) are distributed in sympatry with one of the “*Eulemur fulvus* group” taxa [45]. Loud calls or “croaks” in eulemurs are long and noisy vocalizations that are used during intergroup encounters and as alarm or group cohesion calls [46, 47]. The acoustic structure of *Eulemur* loud calls shows considerable variation, with subtle differences between loud calls of species belonging to the “*Eulemur fulvus* group”, but pronounced acoustic differences between loud calls of members of the “*Eulemur fulvus* group” and the other five members of the genus [44]. Thus, diversification of acoustic signals of *Eulemur* species occurring in allopatry is not pronounced, whereas sympatric species differ, suggesting that the need for reliable species recognition may have favored acoustic diversification.

Accordingly, we predicted that in response to playback experiments, eulemurs do not discriminate (operationalized as time spent looking towards the speaker) between their own loud calls and those of allopatric species, but between their own and loud calls of sympatric congeners. If, however, diversification of acoustic signals is the result of genetic drift, we predicted that eulemurs do not discriminate between loud calls of genetically closely related congeners, but between loud calls of more distantly related congeners. Finally, as heterospecific mating is more costly for females because they invest more in reproduction than males [48, 49], females should respond stronger to these loud calls than males.

Redfronted lemurs are an interesting model species to evaluate the relative importance of different evolutionary pressures in shaping species-specific acoustic signals because this species has a disjunct distribution, with sub-populations occurring in western dry deciduous forests and eastern mountain rain forests (Fig. 4). Whereas *E. rufifrons* populations in the east are sympatric with a congeneric species (*E. rubriventer),* western populations have no sympatric congener. In addition, *E. rufifrons* and *E. rubriventer* produce loud calls during interspecific group encounters (Rakotonirina pers. obs). The acoustic differences between *E. rubriventer* and *E. rufifrons* are much more pronounced than between more closely related species [44]. A previous study indicated no acoustic difference between eastern and western populations, suggesting that there might be no habitat effect on acoustic signals of the two populations of *E. rufifrons* [44]. Since western *E. rufifrons* do not occur in sympatry with *E. rubriventer* but eastern populations do, we predicted different responses to the respective loud calls in each population. Accordingly, western *E. rufifrons* should not discriminate between their own calls and those of *E. rubriventer*, whereas eastern redfronted lemurs should do so.

## Results

### Responses of redfronted lemurs at Kirindy Forest (KF)

**Fig. 1:**
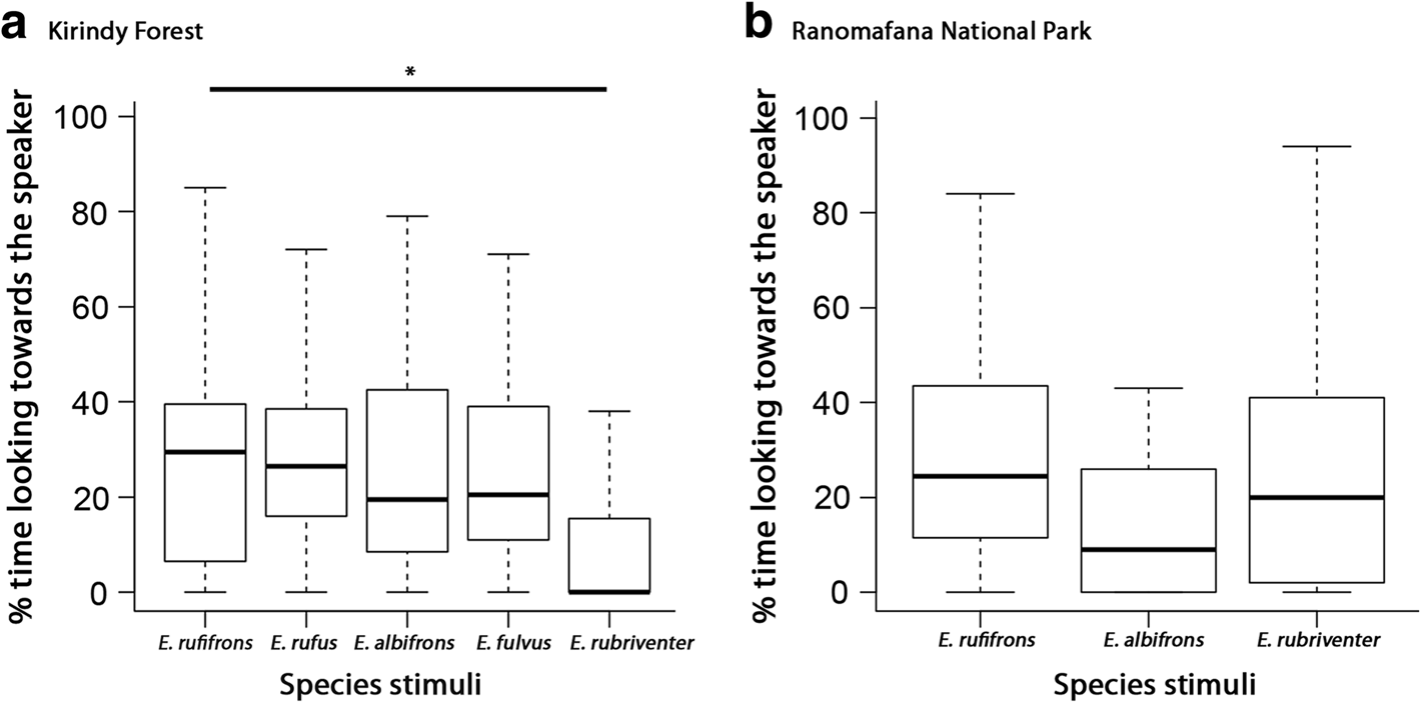
**a**, **b** Boxplot of the percentage of time spent looking towards the speaker of *Eulemur rufifrons* in (**a**) Kirindy Forest and (**b**) in Ranomafana National Park in response to playbacks of loud calls from different congeneric species. Depicted are the median (*black bars*), interquartile range (*boxes*) and ranges (*whiskers*)

The percentage of time spent looking towards the speaker during the first minute following the onset of a playback differed significantly among stimuli (Table 1, LMM, Χ^2^ = 16.64, *p* = 0.005). Specifically, *E. rufifrons* spent less time looking towards the speaker after the presentation of loud calls of the genetically more distantly related *E. rubriventer* (Fig. 1a). There was no sex difference in the percentage of time spent looking towards the speaker after presentation of the different playback stimuli (Table 1). However, the percentage of time spent looking towards the speaker was significantly influenced by the genetic distance between the species (Table 1, LMM, Χ^2^ = 16.15, *p* < 0.001).

**Table 1:** Parameter estimates for the Linear Mixed Models (LMM) on the influence of the different playback stimuli and the genetic distance between species on the percentage of time spent looking towards the speaker for redfronted lemurs tested at Kirindy (a, b) and at Ranomafana (c, d)

	Model	Response variable	Random factors	Fixed factors	Estimate	SE	*P*-value
a	LMM	Percentage of time spent looking towards the speaker	individual identity	intercept	0.56	0.08	<0.001
				*E. rufus*	0.02	0.11	0.81
				*E. albifrons*	−0.02	0.11	0.85
				*E. fulvus*	0.01	0.11	0.91
				*E. rubriventer*	−0.33	0.11	0.003
				sex	−0.07	0.68	0.28
b	LMM	Percentage of time spent looking towards the speaker	individual identity	intercept	0.60	0.05	<0.001
				genetic distance	−0.08	0.02	<0.001
				sex	−0.07	0.07	0.29
c	LMM	Percentage of time spent looking towards the speaker	individual identity	intercept	0.28	0.1	<0.001
				*E. rubriventer*	0.12	0.11	0.09
				*E. albifrons*	0.19	0.11	0.49
				sex	0.09	0.11	0.42
d	LMM	Percentage of time spent looking towards the speaker	individual identity	intercept	0.39	0.09	<0.001
				genetic distance	−0.001	0.02	0.97
				sex	0.07	0.11	0.52

### Responses of redfronted lemurs in Ranomafana National Park (RNP)

*Eulemur rufifrons* at RNP did not differ in the average percentage of time spent looking towards the speaker during the first minute following the onset of a playback between the three different playback stimuli of *E. albifrons*, *E. rubriventer* and *E. rufifrons* (Fig. 1b, Table 1, LMM, Χ = 3.49, *p* = 0.321). There was also no sex difference in time spent looking towards the speaker after presentation of the different playback stimuli (Table 1). The percentage of time spent looking towards the speaker was not influenced by the genetic distance of the two species (Table 1, LMM, Χ^2^ = 0.46, *p* = 0.79).

### Comparison between redfronted lemurs at KF and RNP

**Fig. 2:**
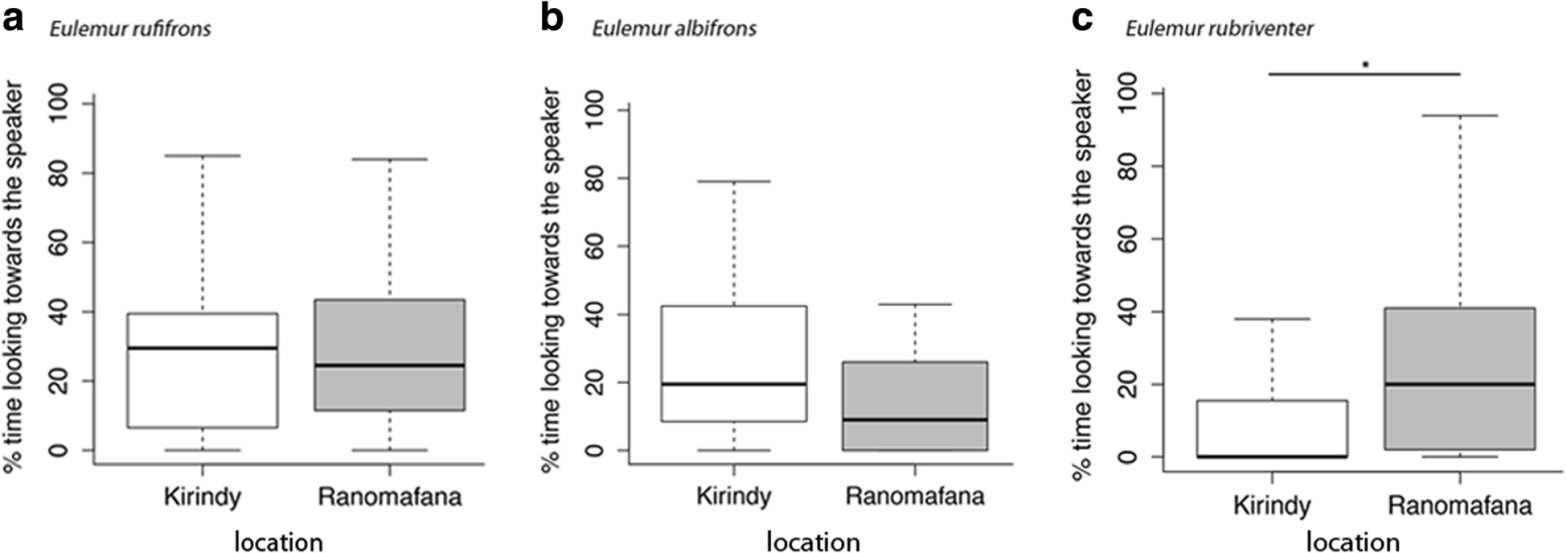
**a**, **b**, **c** Boxplot of time spent looking towards the speaker after presentation of playbacks of **a** *E. albifrons*, **b** *E. rufifrons* and **c** *E. rubriventer* in KF (*white*) and RNP (*grey*). Represented are the median (*black bars*), interquartile range (*boxes*) and range (*whiskers*)

The comparison of looking responses between redfronted lemurs from both populations revealed no significant differences in time spent looking towards the speaker after the presentation of their own species loud calls (Mann Whitney *U* test, *p* = 0.993) and loud calls of *E. albifrons* (Mann Whitney *U* test, *p* = 0.132)*.* However, redfronted lemurs at RNP spent significantly more time looking towards the speaker after presentation of the sympatrically occurring *E. rubriventer* than redfronted lemurs at KF, which do not occur sympatrically with *E. rubriventer* (Mann Whitney *U* test, *p* = 0.026, Fig. 2a, b and c).

### Genetic and acoustic distances

**Fig. 3:**
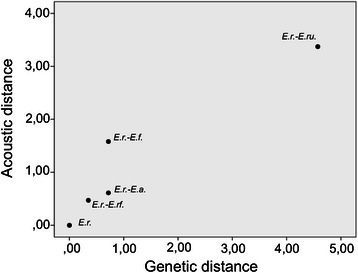
Acoustic distance vs. genetic distance between *E. rufifrons* and the *Eulemur* species used as stimuli. Each *dot* represents acoustic distance vs. genetic distance of one species pair. *E.r.*: *E. rufifrons*, *E.r.-E.rf.*: *E. rufifrons - E. rufus*, *E.r.-E.a*.: *E. rufifrons - E. albifrons*, *E.r.-E.f.*: *E. rufifrons - E. fulvus*, *E.r.-E.ru.*: *E. rufifrons - E. rubriventer*

The genetic distance of the five species correlated positively with their acoustic distance (Spearman rank: rho = 0.98, *p* = 0.005; Fig. 3).

## Discussion

This study investigated the ability of *Eulemur rufifrons* to discriminate between conspecific and heterospecific loud calls. In KF, *E. rufifrons* did not discriminate between loud calls of closely related *E. albifrons*, *E. fulvus* and *E. rufus*. However, they discriminated between their own loud calls and those of *E. rubriventer,* as demonstrated by the shorter time spent looking towards the speaker. In RNP, *E. rufifrons* also did not discriminate between their own loud calls and those of the closely related *E. albifrons* but also not between their own calls and those of the more distantly related *E. rubriventer*. However, redfronted lemurs at RNP spent on average more time looking towards the speaker after presentations of *E. rubriventer* loud calls than did *E. rufifrons* in KF.

### Species recognition and sexual selection

Vocalizations in numerous species of animals, including frogs, insects, birds and primates, are considered to be reliable source for the taxonomic delineation of subspecies or species [4, 20, 32, 50]. However, taxonomic decisions based on difference in vocalizations rarely consider the behavioral reactions of animals to acoustic cues and whether differences measured in vocalizations between subspecies and species are meaningful in terms of reproductive isolation for the taxa in question. Our study showed that differences among loud calls measured in previous studies between closely related eulemur species [44] are apparently meaningless for the animals in terms of a potential reproductive barrier. We therefore suggest that taxonomic studies should investigate several traits and consider also the behavioral responses of the animals under study to traits supposedly involved in reproductive isolation.

The responses of females and males during all playback experiments did not differ from each other in time spent looking towards the speaker. Because females are known to invest more into reproduction than males, and heterospecific mating might be more costly for them [48, 51], we predicted that they should pay more attention to the loud calls and show stronger responses than males. In species where loud calls are also used in the mating context, such as in gibbons [34] or langurs [35], sexual selection might have driven the diversification of calls. However, differences seen between loud calls of *E. rufifrons* and closely related species are obviously not strong enough to contribute to reproductive isolation at least in the “*Eulemur fulvus* group”. In fact, several *Eulemur* species also form viable hybrid populations in some areas in Madagascar [52, 53], even among species exhibiting strong acoustic differences in their loud calls. Acoustic signals seem therefore not be used for avoidance of heterospecific mating in eulemurs, and it seems rather unlikely that call diversification evolved via sexual selection.

### Species recognition and natural selection

Differences between loud calls of the “*Eulemur fulvus* group” seem not to be strong enough that *E. rufifrons* showed differentiated responses after presentation of their own loud calls and those of closely related species. Natural selection and habitat differences therefore seem unlikely to be responsible for the divergence of acoustic signals in eulemurs. There are several *Eulemur* species occurring in similar habitats along the east coast as well as along the west coast (see [45]). Acoustic differences are not stronger between eastern and western species than between species occurring only in the east or only in the west [44]. And, there is also no difference between loud calls of the eastern and the western *E. rufifrons* populations [44] although the same species occurs in different habitats with different ecologies [54]. Moreover, *Eulemur* species occurring in sympatry show the strongest acoustic differences in loud calls despite inhabiting the same habitat and being exposed to similar natural selection pressures [44]. This effect is also evident in our study species because *E. rufifrons* and *E. rubriventer* in RNP show strong acoustic differences. Therefore, natural selection and habitat differences seem unlikely to have played a role in the diversification of acoustic signals in *E. rufifrons*.

### Species recognition and genetic drift

Finally, it is likely that the observed call divergence is mostly influenced by genetic drift. The fact that differences between loud calls of closely related eulemurs are rather small and calls get more distinctive as genetic distance between taxa increases [44], suggests an influence of genetic drift. Although our sample size is rather small, the acoustic and genetic distances correlated positively among the *Eulemur* species investigated in this study. *E. rufifrons* in both populations did not distinguish between calls of closely related species. Since closely related *Eulemur* taxa diverged more recently, genetic drift might not have yet produced strong differences between loud calls to be recognized. In contrast, a recent playback study on two subspecies of saddle-back tamarins (*Saguinus fuscicollis nigrifrons* and *S. f. lagonotus*) revealed that *Saguinus fuscicollis nigrifrons* differentiated between long calls of these two subspecies [55]. However, divergence estimates for these taxa are about 2.9 million years [56], whereas taxa from the “*Eulemur fulvus* group” diverged only during the last 1 million years [44]. Interestingly, in the KF populations, the time spent looking towards the speaker correlated negatively with the genetic distance to the stimulus species, indicating potential effects of genetic drift. Therefore, it seems most parsimonious to conclude at this point that genetic drift played a major role in the diversification of acoustic signals in eulemurs.

### Potential mechanisms involved in species recognition

Acoustic recognition of heterospecific calls has also been documented in other species of mammals occurring in sympatry, for example between redfronted lemurs and Verreaux’s sifakas (*Propithecus verreauxi*) [57], between ring-tailed lemurs (*Lemur catta*) and *P. verreauxi* [58] and between bonnet macaques (*Macaca radiata*) and two species of langurs (*Trachypithecus johnii* and *Semnopithecus entellus*) and *Sambar deer* [59]. Those studies underline the importance of experience and learning for the ability to recognize heterospecific calls for sympatric species and might explain why *E. rufifrons* in this study responded more strongly to loud calls of sympatric *E. rubriventer* in RNP than in KF. Therefore, our results suggest that in *E. rufifrons* in RNP learning may play a role in recognizing heterospecific calls. As *E. rufifrons* and *E. rubriventer* occur sympatrically at RNP, *E. rufifrons* might have paid more attention to loud calls of *E. rubriventer* because they indicate the presence of a food competitor [60, 61]. In fact, experiments were conducted mostly during guava fruiting season and animals of both species were observed feeding from the same resources (personal obs., see also [61]). It is also known that in some species of primates territorial confrontations may occur with neighboring groups of different species and that vocal signals such as loud calls may be used in such contexts in order to defend mates or resources (e.g. between saddle-back tamarins (S*aguinus fuscicollis avilapiresi*) and red-capped moustached tamarins (*Saguinus mystax pileatus*) [62]).

### Other signals for species recognition in eulemurs

Primates and other animals use different signals for communication, and the use of species-specific signals for species recognition has already been demonstrated by several authors [6, 10, 11, 14, 23–25]. However, only few studies have investigated the role of species-specific signals in lemurs [22, 43], even though they represent endpoints of recent adaptive radiations. Whereas species recognition based on olfactory cues has been demonstrated in true lemurs (*Eulemur sp*. [23]) only one study analyzed the role of visual species-specific signals (facial features) in eulemurs [28]. Our study tested the ability of redfronted lemurs to recognize conspecifics from heterospecifics via acoustic signals, suggesting that acoustic signals apparently play a less important role for eulemurs in species recognition. However, *Eulemur* species exhibit a wide variety in terms of facial color patterns and especially males, with the exception of *E. rufifrons* and *E. rufus* (see [45, 63]), show colorful and pronounced facial hair patterns that could serve as species-specific visual signals. We therefore suggest that future studies on species recognition using visual signals may provide important insights into the relative importance of either olfactory, acoustic or visual signals in species recognition of eulemurs.

## Conclusions

We conclude that *E. rufifrons* are not able to discriminate between loud calls of closely related species living in allopatry and that responses to more distantly related congeners are likely to be modulated by experience. *E. rufifrons* at KF discriminated between loud calls of them and their own calls, whereas *E. rufifrons* at RNP did not. Because members of the two study populations responded differently to these calls, we suggest that experience, presumably based on learning, may have modulated the response of the RNP population to calls of *E. rubriventer*, which acts as a food competitor there. In addition, species differences in loud calls are likely partly the result of genetic drift. Since closely related *Eulemur* taxa diverged only recently, genetic drift might not have yet produced strong differences between loud calls to be recognized, suggesting that these calls are less important for species recognition in these cathemeral primates. Thus, playback experiments are important to understand whether differences between acoustic signals used for species delimitation are also used by the animals themselves to discriminate between conspecific and heterospecific calls.

## Methods

### Study sites

**Fig. 4:**
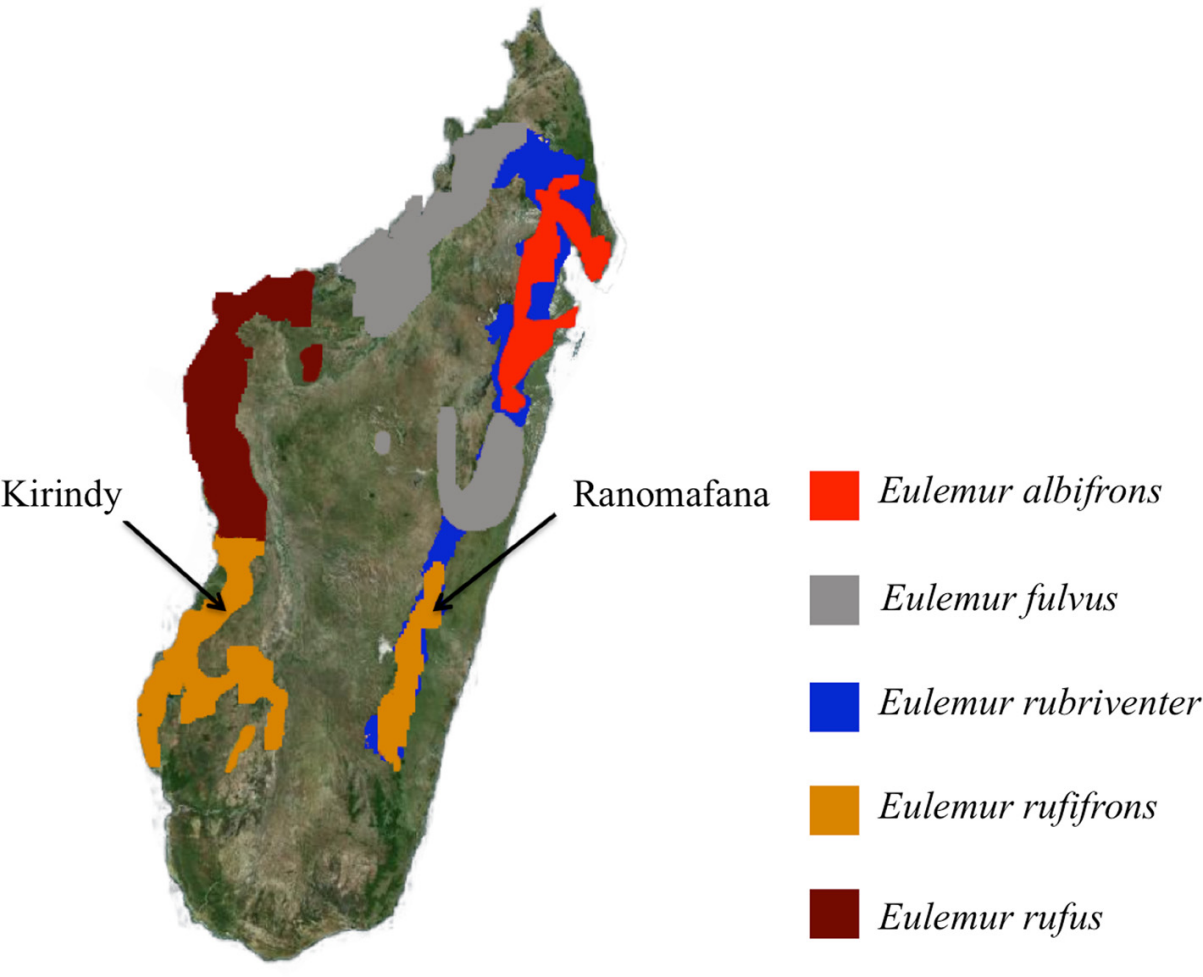
Map of Madagascar with distribution of *Eulemur* species used as stimuli for playback experiments and locations of field sites

Playback experiments were conducted at two sites in Madagascar: Kirindy Forest (KF) and Ranomafana National Park (RNP) (Fig. 4). At KF, *Eulemur rufifrons* have been individually marked as part of a long-term study [64, 65], and we studied 16 individuals (eight females and eight males) from four groups. At RNP, we studied 21 individuals (11 females and ten males) from seven groups that were distinguished by their size, sex ratio and home range location. We recognized individuals through earmarks, scratches or distinctive fur coloration.

### Playback stimuli and design

**Fig. 5:**
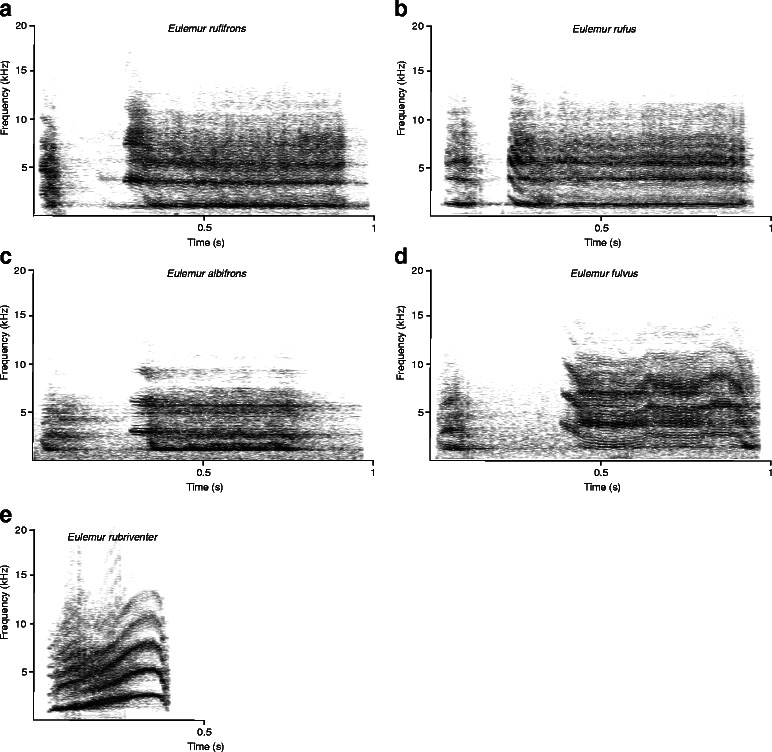
Spectorgrams of loud calls of: **a** *E. rufifrons*, **b** *E. rubriventer,* **c** *E. albifrons.* **d** *E. fulvus*, and **e** *E. rufus*

Loud calls (croaks) used as playback stimuli were recorded as responses to playback experiments with conspecific loud calls in wild populations of *E. albifrons*, *E. fulvus*, *E. rubriventer*, *E. rufifrons* and *E. rufus* as part of an earlier study ([44], Fig. 5, see also Additional files 1, 2, 3, 4, 5). Recordings were made with a Marantz solid-state recorder PMD 660 (frequency response 40–20.000 Hz) and a Sennheiser directional microphone K6 power module and ME66 recording head (frequency response 40–20.000 Hz) with a MZ W66 pro windscreen. Because *E. rufifrons* usually produces bouts of loud calls in territorial contexts, each playback stimulus was repeated twice with intervals of 5 s silence in between, using Cool Edit 2000 (Syntrillium Phoenix, AZ). The sound pressure level of all playback stimuli was adjusted to 34 ± 3 dB using Cool Edit and broadcast with the same volume settings at the loud speaker. Playback stimuli were presented with a Marantz solid-state recorder PMD 660 connected to a loud speaker (Davidactve, Visonik) hidden in the vegetation at a distance of 10 m behind a focal animal, so that the individual looking towards the speaker had to look in the opposite direction of the researcher, who was positioned at a distance of about 7 m in front of the focal subject to video-tape its response.

We used the following 5 stimuli for playback experiments in the KF population: loud calls of *E. albifrons*, *E. fulvus*, *E. rubriventer*, *E. rufifrons* and *E. rufus* (Fig. 5, Table 2). In the RNP population, the number of playback stimuli was reduced from 5 to 3 because some of the groups at RNP could not be located on a regular basis. We therefore presented *E. rufifrons* at RNP only loud calls of their own species as well as calls of *E. albifrons* and *E. rubriventer* (Table 2). In both populations, we used as heterospecific playback stimuli the same calls, however, as conspecific playback stimulus we used calls that were recorded in the respective population (Kirindy or Ranomafana). Since in earlier playback studies with subjects from the population in Kirindy Forest focal subjects did not respond to controls (loud calls from chacma baboons or the song from a local parrot [47, 57]), we refrained from using such a control in the current study because of the low response and the logistical efforts for every single playback are enormous – especially in the rain forest.

**Table 2:** Number of individuals tested for each playback stimulus in both populations

	Population	
Species of playback stimulus	Kirindy Forest	Ranomafana National Park
*Eulemur rufifrons*	*N* = 16	*N* = 16
*Eulemur albifrons*	*N* = 16	*N* = 17
*Eulemur rubriventer*	*N* = 16	*N* = 17
*Eulemur fulvus*	*N* = 16	
*Eulemur rufus*	*N* = 16	

Playbacks were conducted only with animals that were engaged in relatively quiet activities, such as resting or grooming. To avoid pseudo-replication, we used loud calls from a different individual for each playback experiment, and subjects were tested with each stimulus in a randomized but counter-balanced order. Each playback stimulus was tested only once every 2^nd^ day per group. Subjects’ responses to the playback stimuli were recorded with a SONY digital video camera briefly before and 1 min after the onset of each playback experiment. Based on these video-recordings we measured the time the animal spent looking towards the speaker (looking direction within 45° angle to the direct line of sight towards the loud speaker, see Additional file 6) and time spent looking around in other directions after the onset of the playback stimulus, and we calculated the percentage of time spent looking towards the speaker from the total time spent looking around. Video analyses were conducted with a frame-by-frame analysis with a resolution of 30 frames/s using Adobe Premiere Elements (12.0). 10 % of all experiments were scored by a second observer, naive to the research question. The Interclass Correlation Coefficient was very good with ICC = 0.97.

### Statistic analyses

Linear mixed models (LMM) were used to test for differences in the percentage of time spent looking towards the speaker of redfronted lemurs in response to different playback stimuli in both populations respectively using lmerTest package in R [66]. Percentage of time spent looking towards the speaker was arcsine-squareroot transformed and fitted as response. Playback stimulus and sex were fitted as fixed factors and individual identity as random factor. LMMs were also used to examine whether genetic distances between species influenced the percentage of time spent looking towards the speaker, with the latter variable fitted as response, genetic distance and sex as fixed factors and individual identity as random factor. To test for differences in responses of *E. rufifrons* to loud calls of *E. albifrons*, *E. rubriventer* and *E. rufifrons* between the two populations (KF and RNP), we conducted a Mann-Whitney *U* test.

To examine the relationship between genetic distance and acoustic signal divergence, we calculated the Euclidian distance between each pair of species on the basis of the group centroids revealed by a discriminant function analysis calculated in SPSS [44]. The function cophenetic.phylo of the R package APE 3.0–11 was used to calculate pairwise genetic distances between pairs of tips from a phylogenetic tree using its branch length, using the *Eulemur* species tree published by Markolf et al. [44]. Since both populations of *E. rufifrons* do not differ genetically [44], they were combined for this analysis. Acoustic and genetic distances were then subjected to a Spearman’s rank correlation. All analyses were conducted in R version 3.1.2.

## Ethics approval and consent to participate

Not applicable.

## Consent for publication

Not applicable.

## Availability of data

The data will be made available in a public database (dryad.org) prior to publication.

## Additional files

Additional file 1: Audio file of one loud call of *Eulemur rufifrons*. (WAV 3750 kb) Additional file 2: Audio file of one loud call of *Eulemur rufus*. (WAV 3730 kb) Additional file 3: Audio file of one loud call of *Eulemur albifrons*. (WAV 3746 kb) Additional file 4: Audio file of one loud call of *Eulemur fulvus*. (WAV 3744 kb) Additional file 5: Audio file of one loud call of *Eulemur rubriventer*. (WAV 3636 kb) Additional file 6: Average looking duration towards the speaker after each playback stimulus in the populations at Kirindy Forest and Ranomafana National Park. (PDF 110 kb)
