# Supplementary material for: The role of acoustic signals for species recognition in redfronted lemurs (Eulemur rufifrons)
Source: BMC Evol Biol. 2016 May 12;16:100. doi: 10.1186/s12862-016-0677-1 (PMC4866039; doi:10.1186/s12862-016-0677-1)
Supplement: Additional file 6: — Average looking duration towards the speaker after each playback stimulus in the populations at Kirindy Forest and Ranomafana National Park. (PDF 110 kb) [file 12862_2016_677_MOESM6_ESM.pdf]

Additional files

Additional file 6: Average looking duration towards the speaker after each playback stimulus in the populations at Kirindy Forest and Ranomafana National Park.

Species used as playback stimuli	Location	Looking duration towards the speaker (average)
Eulemur albifrons	Kirindy	15.80 (±14.36)
	Ranomafana	9.61 (±11.00)
Eulemur rubriventer	Kirindy	4.56 (±6.93)
	Ranomafana	13.89 (±15.49)
Eulemur rufifrons	Kirindy	14.95 (±12.79)
	Ranomafana	16.73 (±15.53)
